# Awareness and Work Changes of Dental Hygienists about Newly Covering Health Insurance Benefit for Scaling

**Published:** 2017-04

**Authors:** Yu-Ri CHOI, Youn-Soo SHIM, Sun-Ok JANG, Seoul-Hee NAM, Gyeong-Soon HAN

**Affiliations:** 1. Dept. of Dental Hygiene, Hallym Polytechnic University, Chuncheon, Republic of Korea; 2. Dept. of Dental Hygiene, College of Health Science, Sunmoon University, Asan, Republic of Korea; 3. Dept. of Dental Hygiene, Kangwon National University, Samcheok, Republic of Korea; 4. Dept. of Dental Hygiene, College of Health Science, Gachon University, Incheon, Republic of Korea

## Dear Editor-in-Chief

In the South Korea, the national health insurance (NHI) system has been established to provide everybody with health care services to promote national health (1–2). Health Insurance Review & Assessment Service analyzed the review and decision data of five years from 2008 to 2012 to find out about treatment and treatment cost for “gingivitis and periodontal diseases” (3). The number of patients who received treatment for these diseases showed an increase of approximately 1.70 million (25.3%) from 6.73 million in 2008 to 8.43% in 2012. The yearly mean increase rate stood at 4.6%. There was an increase about 196.6 billion won (66.2%) in total treatment expenses over the five years, from about 297.0 billion won in 2008 to 493.6 billion won in 2012, and the annual average increase rate stood at 10.7% (3). Therefore, adding dental scaling to the coverage list of the NHI system was discussed, which makes it possible to prevent periodontal diseases by eliminating dental deposits including dental calculus (4, 5).

The purpose of this study was to examine the awareness of dental hygienists in charge of scaling in dental institutions about the advantages of the addition of scaling to the coverage list of the NHI system, problems with it and subsequent changes in their work in an effort to provide some information on this matter.

This study was conducted with the approval of the Institutional Review Board of a university (IRB No. 1044396-201508-HR-047-0).

A self-administered survey was conducted on 340 selected dental hygienists in 2015. The questionnaire used in this study consisted of six on the scaling of the NHI system and eight on changes in the duties of dental hygienists. The collected data were analyzed by a statistical package SPSS 19.0 (IBM Co., Armonk, NY, USA). Frequency analysis, t-test and one-way ANOVA were carried out, and stepwise multiple regression analysis was conducted to make a factor analysis.

As for awareness on the advantages of the addition of scaling to the coverage list of the NHI system, they perceived that it was effective at preventing periodontal diseases and contributed to raising awareness of oral health care. Concerning problems with it, they pointed out time constraints that stemmed from the increase in the number of patients. Only 14.2% of dental institutions hired new dental hygienists, whereas, there was an 85.8% increase in the number of scaling patients (Table 1, 2). Consequently, work hours for every kind of duty of the dental hygienists including patient management and preventive duties increased except work hours for explanation on the necessity of scaling. Their overall workload showed a great increase, and it seems urgently required to secure more dental hygienists in accordance with the increase in the number of patients. The patients expressed a little higher satisfaction, but the dental hygienists took a positive view of the addition of scaling though their workload increased. A significant finding shows the professionalism of the dental hygienists. The explainability of the regression model of the factor analysis about their entire workload was 18.9 %. The most related factor was the number of scaling patients, and hours for explanation on the importance of preventive management, hiring new employees and hours for explanation on the necessity of scaling turned out to be related factors as well.

**Table 1:** Health insurance of scaling that advantage, problem, and suggestion (n: 340)

**Characteristics**		**n (%) / M±SD**
Advantage^*^		
Scaling patient increase		107(31.5)
Periodontitis prevention effect		195(57.4)
Easily patient management		32(9.4)
Oral management awareness effect		165(48.5)
Additional clinic cause		35(10.3)
Problem		
Indiscretion clinic		1(0.3)
Accuracy decrease	lack of time due to patient increase	164(48.2)
	Pressure reduction due to patient cost reduction	33(9.7)
None		142(41.8)
Suggestion^**^		
Object age increase		88(25.9)
Medical charge increase		77(22.6)
Increase number		146(42.9)
Number of scaling patient visits		
Decrease or unchanged		48(14.2)
Increase		292(85.8)
Number of dental hygienist employment		
Decrease or unchanged		301(88.5)
Increase		39(11.5)
Satisfaction		
Patient		4.36±0.94
Dental hygienist		4.16±1.24
Change of dental hygienist work		
Explanation time of scaling necessity		2.95±0.74
Explanation time of scaling precaution		3.19±0.63
Importance of explanation time of scaling		3.20±0.61
Patient status screening time		3.12±0.59
Overall workload		3.89±0.69

Overlap response results of *534 persons and **311 persons.

**Table 2:** Stepwise multiple regression related factors of overall workload of dental hygienist

**Independent variables**	**B**	**SE**	**β**	**t**	**P**
Scaling patient	0.527	0.095	0.283	5.554	<0.001
Explanation of prevention clinic importance	0.184	0.055	0.164	2.846	0.005
Number of dental hygienist employment	0.219	0.097	0.115	2.244	0.026
Explanation time of scaling necessity	0.107	0.054	0.115	1.990	0.047
F=3.958(*P*<0.001), R^2^=0.189, adjusted.R^2^=0.179,					
Dependent variables: overall workload					
Excluded variables: explanation time of scaling precaution, patient status screening time					

Although the dental hygienists took a positive view of the addition of scaling to the coverage list of the NHI system, sustained heavy workload is bound to result in detracting from the quality of professional duties. Therefore, it has urgently required to secure personnel in charge of scaling only or to hire more human resources to ensure the successful entrenchment of the scaling service as one of the health insurance benefits.
